# Commencement Exercises

**Published:** 1898-06

**Authors:** 


Secretary.

### COMMENCEMENT EXERCISES.

### McKILLIP VETERINARY COLLEGE.

THE second annual commencement exercises of this college were held in the college auditorium at 8 P.M., March 30, 1898.

The exercises were opened by an invocation by the Rev. E. C. Snyder, after which Prof. L. A. Merillat delivered the "Baccalaureate Address." The "Salutatory" was rendered by Dr. Louis Wagner; "Class History," by Dr. A. C. Spurling; "Class Poem," by Dr. C. B. Davis, of the graduating class. The degree of M.D.V. was conferred upon the following students by President M. H. McKillip: George J. Dandurand, Chicago; Clarence B. Davis, Chicago; James H. Hawke, Chicago; Frederick J. Leith, M.D.C., Chicago; Addison C. Spurling, West McHenry; Louis Wagner, South Chicago, Ill.; William S. Williamson, San Francisco, Cal.; William Wilson, Menomonie, Wisconsin; and W. E. A. Wyman, V.S., Post-Graduate, Charleston, South Carolina. Dr. J. H. Hawke was President and Dr. W. L. Williamson, Secretary of the Class of '98. The benediction closed the exercises. One member failed to obtain the necessary average.

### CHICAGO VETERINARY COLLEGE.

THE fifteenth session of the Chicago Veterinary College terminated with appropriate exercises on March 23d at 2 o'clock in the college auditorium.

The degree of Doctor of Comparative Medicine was conferred on the following gentlemen: George E. Totten, Bement, Ill.; O. O. Wolf, Ottawa, Kan.; Wm. A. Withers, New Orleans, La.; C. G. Warner, Aurora, Ill.; James M. O'Reilley, Merrill, Wis.; James L. Kling, McGrawsville, Ind.; D. A. Piatt, Lexington, Ky.

The trustees' gold medal for highest general average in final examinations was awarded to Dr. G. E. Totten. The prize in theory and practice was awarded to Dr. O. O. Wolf. The prize in anatomy, not having been won by a Senior, was carried over to next year.

The class valedictory was delivered by Dr. O. O. Wolf, and the doctorate by Prof. A. H. Baker. The address of the professor was replete with good advice to the young doctors, giving them some valuable hints as to how to successfully conduct a practice; how to advantageously give expert testimony in court, and dwelt at length on the necessity of sobriety and industry in order to maintain a respectable position in society, to read the professional journals, join their local, State, and national associations, thereby giving themselves a chance to profit by the investigations, observations, and experience of older members of the profession, and wear off the diffidence and narrow-mindedness that always result from non-intercourse with others. He said by way of explanation of the smallness of the graduating class that this was the connecting link between the two- and three-year courses, and under ordinary circumstances there would be no graduating class this year, but on account of allowance for former attendance and attendance at other veterinary colleges there were the few mentioned above, and that what they lacked in numbers they made up in quality, most of them having passed in the honor class, and that the C. V. C. expected to add materially to her reputation through the Class of '98.

He also expressed the opinion that the profession in the West would soon see brighter days; that the depression of the last three years had already given way to marked improvement, which bids fair, in fact, is certain, to continue to improve till in a year or two more the practice will equal, if not exceed, that of 1892. The farmers of the West are in excellent condition financially, having been able to pay their debts during 1897, and are getting rich this year on account of the high prices of grain; and they are breeding all available mares to supply an impoverished horse-market. Good draught-, coach-, and driving-horses are selling now at old-time prices, with every prospect of going much higher long before time enough will have elapsed to grow them. Breeding of horses, he said, in his opinion, would be confined to the farmers hereafter, the big ranches having found it a losing industry; their big horses were not big enough; their drivers were not fine enough, and the inevitable brand that each one carried was so objectionable that they always had to sell cheap, and, unfortunately, pulled the whole race in this country down with them. All of this has stopped, never to return. Veterinarians will profit by this change more than any other profession, as they have suffered more in the past from the great depression.

The veterinary colleges will reap their share of benefit also. The classes will be larger, and more interest will be taken; the students will not feel lonesome in the lecture-room as in the past two or three sessions, on account of the small attendance. There will be no danger of overcrowding the profession for generations to come; the diversified farming that is rapidly developing will in time spread over the entire country, the result of which will be more stock-raising of all kinds, creating in turn an increased demand for veterinarians. The veterinary sun is not setting, but instead will shine brighter than ever after having emerged from the partial eclipse that has existed for the past three years.

